# Relaxant Action of Diclofenac Sodium on Mouse Airway Smooth Muscle

**DOI:** 10.3389/fphar.2019.00608

**Published:** 2019-06-18

**Authors:** Chunfa Chen, Yongle Yang, Meng-Fei Yu, Shunbo Shi, Shuhui Han, Qing-hua Liu, Congli Cai, Jinhua Shen

**Affiliations:** ^1^Institute for Medical Biology, Hubei Provincial Key Laboratory for Protection and Application of Special Plant Germplasm in Wuling Area of China, College of Life Sciences, South-Central University for Nationalities, Wuhan, China; ^2^Department of Molecular Biology, Wuhan Youzhiyou Biopharmaceutical Co. Ltd., Wuhan, China

**Keywords:** airway smooth muscle, BK channels, diclofenac sodium, relaxation, tracheal rings, voltage-dependent Ca^2+^ channels

## Abstract

Diclofenac sodium (DCF) is a nonsteroidal anti-inflammatory drug (NSAID) and is widely used as an analgesic and anti-inflammatory agent. Herein, we found that DCF could relax high K^+^ (80 mM K^+^)-/ACh-precontracted tracheal rings (TRs) in mice. This study aimed to elucidate the underlying mechanisms of DCF-induced relaxations. The effects of DCF on airway smooth muscle (ASM) cells were explored using multiple biophysiological techniques, such as isometric tension measurement and patch-clamping experiments. Both high K^+^- and ACh-evoked contraction of TRs in mice were relaxed by DCF in a dose-dependent manner. The results of isometric tension and patch-clamping experiments demonstrated that DCF-induced relaxation in ASM cells was mediated by cytosolic free Ca^2+^, which was decreased via inhibition of voltage-dependent L-type Ca^2+^ channels (VDLCCs), nonselective cation channels (NSCCs), and Na^+/^Ca^2+^ exchange. Meanwhile, DCF also enhanced large conductance Ca^2+^ activated K^+^ (BK) channels, which led to the relaxation of ASMs. Our data demonstrated that DCF relaxed ASMs by decreasing the intracellular Ca^2+^ concentration via inhibition of Ca^2+^ influx and Na^+^/Ca^2+^ exchange. Meanwhile, the enhanced BK channels also played a role in DCF-induced relaxation in ASMs. These results suggest that DCF is a potential candidate for antibronchospasmic drugs used in treating respiratory diseases such as asthma and chronic obstructive pulmonary disease.

## Introduction

Approximately 300 million individuals suffer from asthma worldwide (World Health Organization, 2001). Asthma is characterized by variable and recurring episodes, such as wheezing, shortness of breath, chest tightness, and coughing (Asher et al., 2006; Lotvall et al., 2011). In most cases, asthma can be controlled using traditional drugs, including corticosteroids (Adams and Jones, 2006; Kowalski et al., 2016), long/short-acting beta-agonists (Black et al., 2009; Morales, 2013), leukotriene receptor antagonists (Stoloff, 2000), and Chinese medicine herbs (Yang et al., 2017). Sometimes, exacerbations still occur and may require emergency room visits, hospitalization, and intensive treatments. This is becoming a large economic, social, and healthcare burden. Therefore, it is urgent to develop new effective drugs to treat asthma.

Ca^2+^ plays important roles in regulating the tone of airway smooth muscles (ASMs) (Somlyo and Somlyo, 1994; Zhang et al., 2014; Wang et al., 2018). The concentration of intracellular Ca^2+^ ([Ca^2+^]_i_) in ASM cells depends on many factors, including intracellular Ca^2+^ release, extracellular Ca^2+^ influx, and Na^+^/Ca^2+^ exchange (Wen et al., 2018; Li and Shang, 2019). Among them, the determinant one is extracellular Ca^2+^ influx, which is regulated by voltage-dependent L-type Ca^2+^ channels (VDLCCs), nonselective cation channels (NSCCs), and Na^+^/Ca^2+^ exchange (Sathish et al., 2011; Rahman et al., 2012). In the clinic, drugs such as long-term beta-2 receptor agonists are used in the management of asthma via decreasing the concentration of [Ca^2+^]_i_, resulting in inactivation of myosin light chain kinase and the relaxation of ASMs (Cazzola et al., 2013).

Diclofenac sodium (DCF) is a nonsteroidal anti-inflammatory drug (NSAID) widely used in the treatment of inflammatory diseases and pain (McNicol et al., 2018). NSAIDs are characterized as the blockers of the cyclooxygenase (COX) isoenzymes, COX1, and COX2 (Arumugam et al., 2013). The anti-inflammatory effect of NSAIDs is via the blockade of COX derived from modulation of chemical mediators, thereby blocking the production of pro-inflammatory prostaglandins by means of chelation (Hawkey, 2001). Meanwhile, DCF-induced inhibition of these two COX enzymes interrupted the conversion of arachidonic acid to eicosanoids (Bosetti et al., 2003), which play a pivotal role in the regulation of the homeostasis of gastrointestinal, renal, and cardiovascular systems. Moreover, DCF has additional action mechanisms, such as the reduction of leukotriene (Ku et al., 1985). Therefore, DCF is the most popular NSAID and is widely used in the treatment of many other diseases (Haley and von Recum, 2018). For example, it has been used in treating osteoarthritis of the knee (Fuller and Roth, 2011), renal colic (Sivrikaya et al., 2003), exercise-induced skeletal muscle damage (O’Grady et al., 2000), and vascular smooth muscle proliferation (Brooks et al., 2003). Previous studies reported that DCF participated in modulation of Ca^2+^ homeostasis via regulating Na^+^/Ca^2+^ exchange (Perez Vallina et al., 1995) and NSCCs, suggesting that DCF has the potential ability of modulating the contraction of ASMs. However, the use of DCF in asthmatic patients should be prudent. Some studies reported that the use of NSAIDs such as DCF may lead to the exacerbated respiratory disease phenotypes (Lo et al., 2016; Tan and Hsu, 2016). Therefore, it is urgent to elucidate the action mechanisms of DCF in asthmatic patients.

Our lab is focusing on developing new drugs for bronchospasm via two methods: screening new ingredients from natural plants and old drugs. DCF is screened as a potential candidate for relaxing precontracted ASMs in an “old drug, new function” way. In the present study, mouse tracheal rings (TRs) and ASM cells were isolated. The tissues/cells were stimulated using high K^+^ (80 mM K^+^) or ACh to mimic the physical state of airway hyperactiveness to evaluate the potential relaxant ability in TRs and elucidate the underlying mechanisms. Our results demonstrate that DCF effectively relaxed ACh/high K^+^-precontracted TRs by inhibiting VDLCC-mediated extracellular Ca^2+^ influx and enhancing the activity of BK channels. These data suggest that DCF is potential new anti-asthma drug candidate.

## Materials and Methods

### Reagents

Unless otherwise stated, all chemicals were purchased from Sigma-Aldrich Corporation.

DCF (Cat No. S43053, HPLC purity ≥ 99%) was purchased from Yuanye Biotechnology Co., Ltd. (Shanghai, China). Nifedipine was dissolved in dimethyl sulfoxide (DMSO) as a stock solution.

### Solutions

The physiological saline solution (PSS) solution comprised the following components (in mM): 135 NaCl, 5 KCl, 1 MgCl_2_, 2 CaCl_2_, 10 glucose, and 10 HEPES (pH 7.4). To generate the high K^+^ solution (80 mM K^+^), the NaCl in the PSS was replaced with an equimolar concentration of KCl. All experiments were conducted at room temperature (22–25°C).

### Animals

Male SPF BALB/c mice (6–8 weeks) were purchased from the Hubei Provincial Center for Disease Control and Prevention (Wuhan, China). Mice were housed in a 12-h light/dark cycle with *ad libitum* access to food and water. All protocols and experiments involving animals were conducted in strict accordance with the guidelines of the Institutional Animal Care and Use Committee of the South-Central University for Nationalities. This study was approved by the Animal Ethical Committee of South-Central University for Nationalities (Approval No. 2017-JHS-1).

### Isometric Tension in Tracheal Rings

Muscle force of TRs was measured as we previously reported elsewhere (Wang et al., 2019). Briefly, mice were sacrificed following intraperitoneal injection of pentobarbital sodium (150 mg/kg). Then, the trachea was cut off and immersed in ice-cold PSS. After removal of connective tissues, the trachea was vertically mounted in a 10-ml organ bath with a 0.3-g preload. The TRs were perfused with oxygenated PSS at 37°C. After a 60-min equilibrium, the TRs were precontracted with 100 μM ACh and washed three times. Following a 30-min rest, the formal experiments started.

### Isolation of Single Airway Smooth Muscle Cells

Single mouse ASM cells were enzymatically isolated as previously reported (Liu et al., 2017; Tan et al., 2017). Briefly, the TRs were isolated as abovementioned and immersed in ice-cold low-Ca^2+^ PSS (LCPSS) composed of 135 mM NaCl, 5 mM KCl, 1 mM MgSO_4_, 10 mM glucose, 10 mM HEPES, and 0.1 mM CaCl_2_ (pH 7.4). After removal of the epithelial cells using a fine forceps, the ASM trips were cut off and incubated in LCPSS containing 1 mg/ml papain, 0.5 mg/ml dithioerythritol, and 1 mg/ml BSA for 20 min at 37°C. Then, the muscle trips were transferred into LCPSS containing 1 mg/ml collagenase H, 1 mg/ml dithiothreitol, and 1 mg/ml BSA for 20 min at 37°C. After three washes in LCPSS, the tissues were triturated using a fire-polished glass pipette to obtain single ASM cells. Single ASM cells were stored in 1.5-ml Eppendorf tubes on ice and used within 4 h.

### Recording of Voltage-Dependent L-Type Ca^2+^ Channel Currents

VDLCC currents were recorded as previously reported with some modifications (Yang et al., 2018; Zhang et al., 2018). Briefly, mouse ASM cells were isolated as described above. VDLCC currents were measured using Ba^2+^ as the charge carrier with an EPC-10 patch-clamp amplifier (HEKA, Lambrecht, Germany). The composition of the pipette solution was (in mM): 130 CsCl, 10 EGTA, 4 MgCl_2_, 4 Mg-ATP, 10 HEPES, and 10 tetraethyl ammonium chloride (pH 7.2). Meanwhile, the bath solution contained (in mM) 105 NaCl, 6 CsCl, BaCl_2_, 11 glucose, 10 HEPES, and 0.1 niflumic acid (NA) (pH 7.4). The holding voltage was −70 mV. Currents were recorded following depolarization for 300 ms from −70 to +40 mV in 10-mV increments every 1 s.

### Recording of K^+^ Currents

To measure the currents of K^+^ channels, the whole-cell recording method was used as described previously (Huang et al., 2017). Briefly, the currents were elicited from a holding potential −80 to +80 mV in 10-mV increments. The composition of the pipette solution was as follows (in mM): 10 NaCl, 125 KCl, 6.2 MgCl_2_, 10 EGTA, and 10 HEPES (adjusted to pH 7.2 with KOH). The bath solution contained (in mM) 150 NaCl, 5.4 KCl, 0.8 MgCl_2_, 5.4 CaCl_2_, and 10 HEPES (adjusted to pH 7.2 with KOH).

### Recording of Single K^+^ Channel Currents

Single BK currents were measured as described previously (Wei et al., 2015; Liu et al., 2018; Qian et al., 2018). Briefly, outside-out patch clamp techniques under symmetrical K^+^ ion concentrations were used to record BK currents. The intracellular solution composition was as follows (in mM): 140 KCl, 1 MgCl_2_, 5 EGTA, 4.37 CaCl_2_, and 10 HEPES (adjusted to pH 7.2 with KOH). The bath solution contained (in mM) 140 KCl, 1 MgCl_2_, 4.9 CaCl_2_, 1 EGTA, and 10 HEPES (adjusted to pH 7.2 with KOH). The digitization rate was 4 kHz and filtered at 1 kHz. All-point amplitude histogram and single channel open probability (Po) were acquired using Clampfit 9 software (Axon Instruments; Foster, CA, USA).

### Measurement of Respiratory System Resistance

Airway reactivity to DCF was measured using a forced oscillation technique as previously described with some modifications (Shalaby et al., 2010). Briefly, mice were weighed and anesthetized by intraperitoneal injection of 10 mg/kg sodium pentobarbital. When the mice reached the desired level of anesthesia, they were tracheostomized using an 18G metal cannula. Then, the mice were placed in a flow-type body plethysmograph and connected with a flexiVent system (SCIREQ, Montreal, PQ, Canada) by an endotracheal cannula. Following the initiation of mechanical ventilation, the mice were subjected to deep lung inflation before the plethysmograph was completely sealed for the rest of the experiments. The mice were ventilated at 150 breaths per minute and tidal volume of 10 ml/kg against a positive end expiratory pressure of 3 cm H_2_O. The mice were exposed to aerosol ACh at different concentrations (43, 86, 172, 343, 516, and 685 mM, each for 30 s). Respiratory system resistance (Rrs) was acquired for 120 s for ACh/DCF at each concentration and calculated using flexiVent software.

### Statistical Analysis

Values are expressed as the means ± SEM. Statistical analysis and significance were measured with Student’s t-test or a one-way analysis of variance (ANOVA) using Origin 9.0 software (OriginLab, Northampton, USA). *P* < 0.05 was considered statistically significant.

## Results

### Diclofenac Sodium Relaxes High K^+^-Precontracted Mouse Tracheal Rings

**Figure 1:**
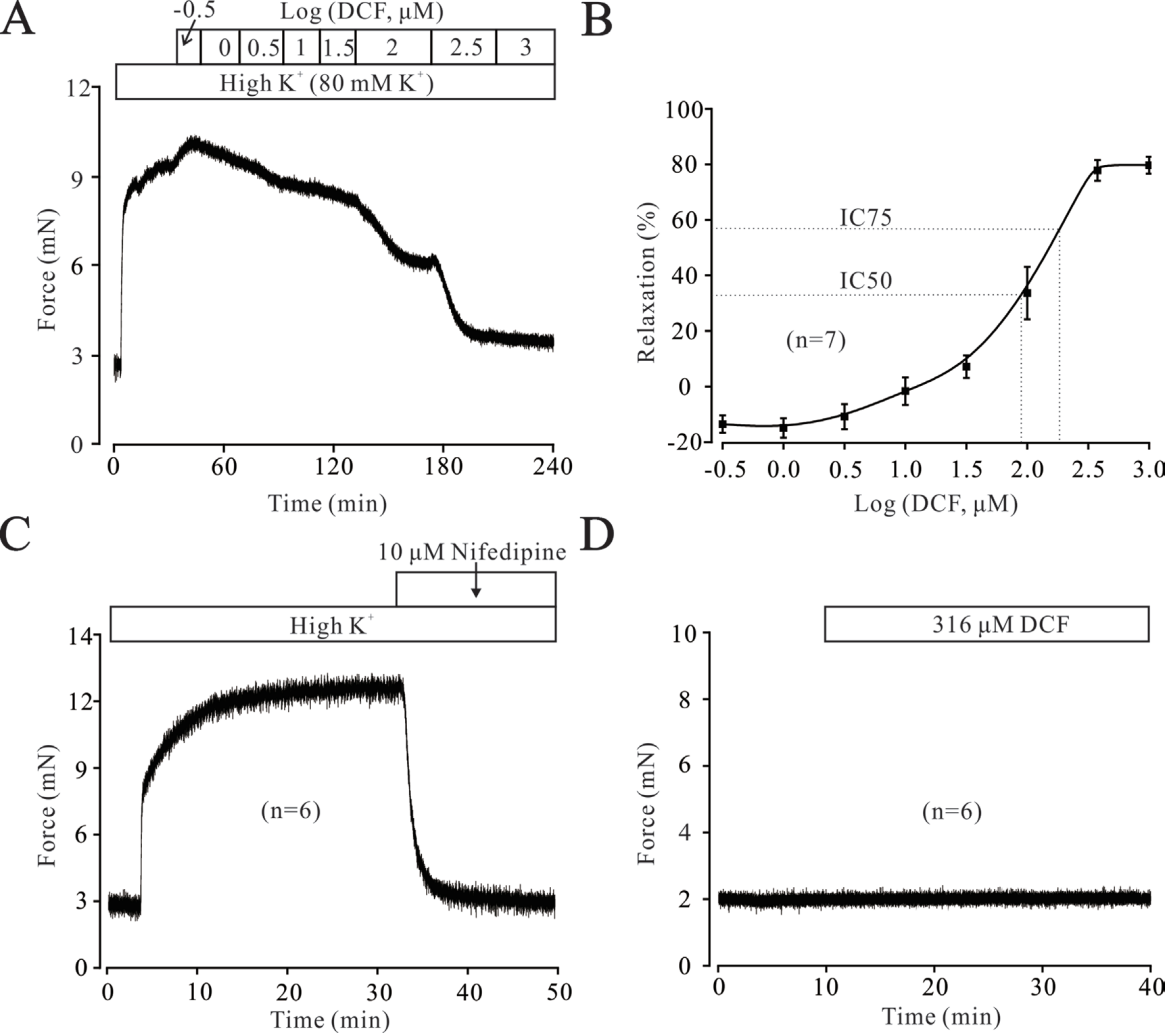
Diclofenac sodium (DCF) relaxes high K^+^-precontracted mouse tracheal rings (TRs). **(A)** High K^+^ induced a contraction in a mouse TR. When the contraction reached the plateau level, DCF was added, and the precontracted TR was relaxed in a dose-dependent manner. **(B)** Dose-response curve of precontracted TRs to DCF (n = 7). **(C)** High K^+^ elicited a stable large contraction in a mouse TR, which was completely inhibited by nifedipine, a selective inhibitor of VDLCCs (n = 6). **(D)** The addition of 316 μM DCF did not alter the basal tone of mouse TRs (n = 6). These results demonstrate that DCF can effectively relax TRs by inhibiting VDLCCs.

To explore the potential relaxation ability of DCF in ASMs, we first detected the effects of DCF in precontracted mouse TRs. As shown in **Figure 1A and B**, DCF relaxed high K^+^-induced contraction in a dose-dependent manner. The maximal relaxation produced by DCF was 80.5% (IC75 = 298 μM). Meanwhile, high K^+^-elicited contraction was completely inhibited by a specific inhibitor of voltage-dependent L-type Ca^2+^ channels, nifedipine (**Figure 1C**). Moreover, DCF at the maximal concentration of 316 μM did not alter the basal tone of mouse TRs (**Figure 1D**). These results demonstrate that DCF can relax precontracted TRs without harmful effects.

### Diclofenac Sodium Inhibits High K^+^-Induced Ca^2+^ Influx

**Figure 2:**
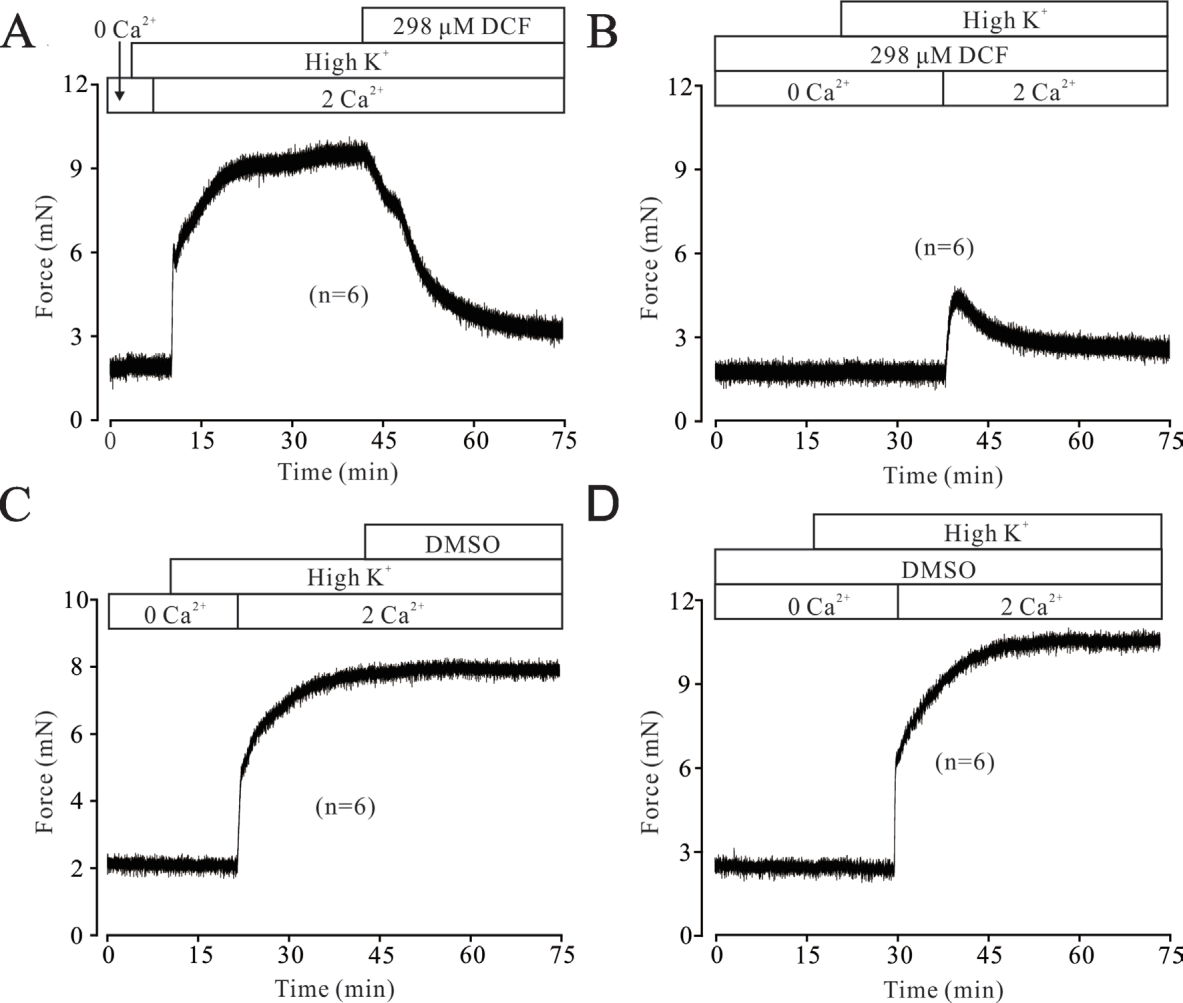
DCF inhibits high K^+^-induced Ca^2+^ influx. **(A)** Under 0 Ca^2+^ conditions (0 Ca^2+^ +0.5 mM EGTA), high K^+^ failed to induce a contraction in mouse TRs. After the restoration of 2 mM Ca^2+^, high K^+^ induced a stable large contraction, which was inhibited by 298 μM DCF (n = 6). **(B)** Under the same condition as in **(A)**, the solvent of DCF, dimethyl sulfoxide (DMSO), was used to replace DCF. However, DMSO failed to induce a relaxation in high K^+^-precontracted TRs (n = 6). **(C)** With the presence of 298 μM under 0 Ca^2+^ conditions, the addition of high K^+^ failed to induce a contraction in mouse TRs. Following the addition of 2 mM Ca^2+^, a relatively small contraction occurred, which was gradually inhibited by DCF (n = 6). **(D)** Under the same conditions as in **(C)**, DMSO was used to replace DCF. After the restoration of 2 mM Ca^2+^, a large stable contraction occurred, which was not inhibited (n = 6). These results suggest that DCF relaxes TRs via inhibiting Ca^2+^ influx.

High K^+^ resulted in membrane depolarization, which in turn activated VDLCCs, leading to extracellular Ca^2+^ influx and triggering the contraction of ASMs. To uncover the underlying mechanisms of DCF-induced relaxation in TRs, we then studied the effects of DCF on Ca^2+^ influx. As shown in **Figure 2A**, without the presence of extracellular Ca^2+^, high K^+^ failed to induce a contraction in mouse TRs. However, after the restoration of 2 mM Ca^2+^ in bath solution, high K^+^ elicited a large, stable contraction, which was mostly inhibited by 298 μM DCF. Similarly, preincubation of TRs with DCF also mostly abolished the high K^+^-induced contraction under the conditions of restoring extracellular Ca^2+^ from 0 to 2 mM (**Figure 2B**). The relaxation effects of DCF in mouse TRs were not due to the solvent for DCF, DMSO (**Figures 2C and D**). These data indicate that DCF-induced relaxation in TRs contributed to the blockade of Ca^2+^ influx by inhibiting VDLCCs.

### Diclofenac Sodium Blocks Voltage-Dependent L-Type Ca^2+^ Channel Currents

**Figure 3:**
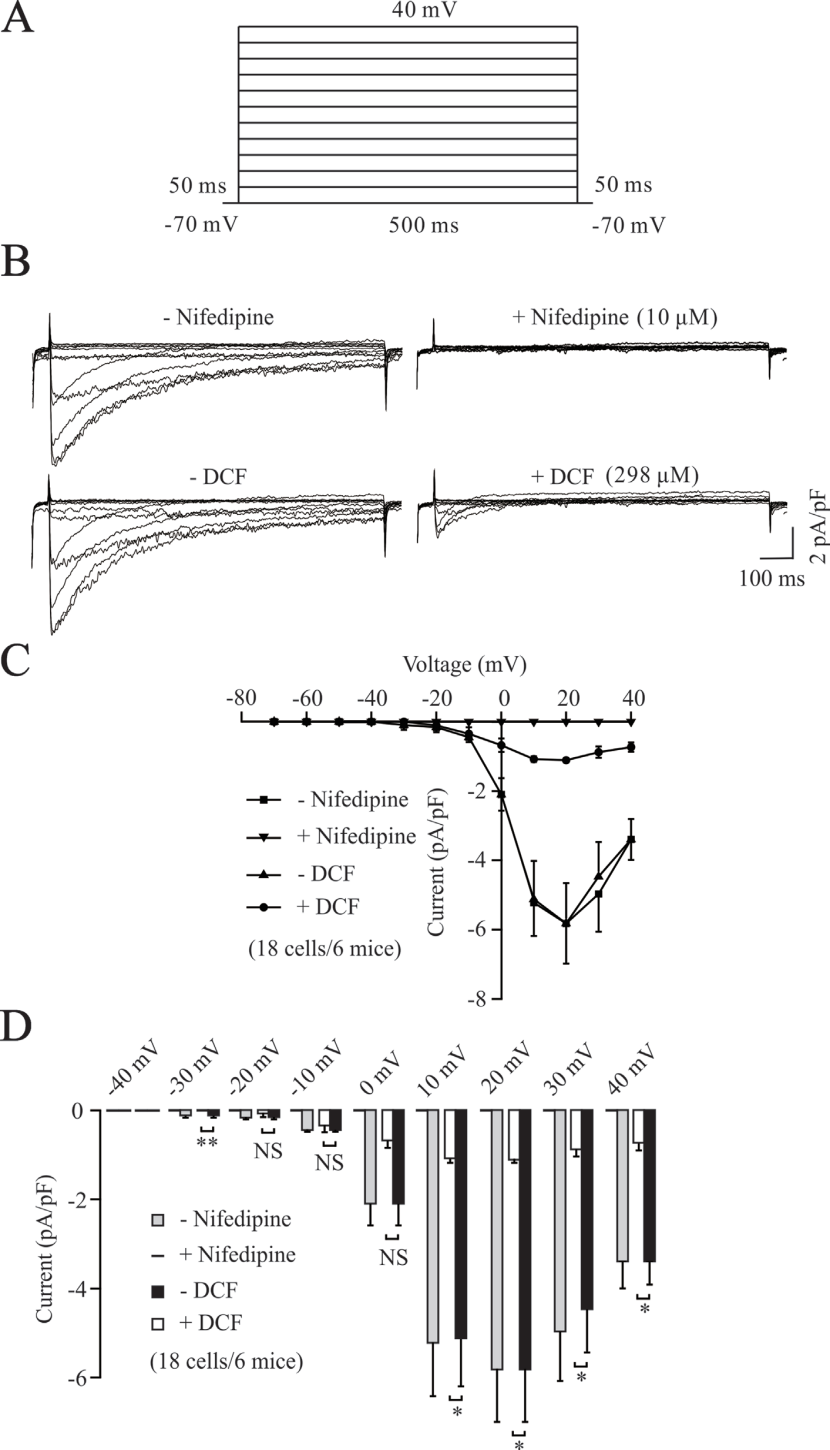
DCF blocks VDLCC currents. **(A)** The patch-clamping technique was used to record the whole-cell VDLCC currents in single ASM cells. The voltage ranged from −70 to + 40 mV. **(B)** Representative VDLCC currents recorded in the absence and presence of 10 μM nifedipine, a selective inhibitor of VDLCCs (upper panel) or 298 μM DCF. **(C)** Current–voltage relationships for VDLCCs obtained in **(B)** (18 cells/6 mice). **(D)** Statistical analysis of the inhibitory effects of DCF on VDLCC currents based on **(B)** (paired t-test). *: *P* < 0.05; **: *P* < 0.01. These results demonstrate that DCF can partly block VDLCC currents.

To further elucidate the relaxant effects of DCF in TRs, we performed the whole-cell patch clamp technique to record the changes of VDLCCs. As shown in **Figure 3A and B**, VDLCCs were elicited by step voltage depolarization from −70 to +40 mV in an increment of 10 mV, and completely blocked by 10 μM nifedipine, a specific inhibitor of VDLCCs. Then, we observed the effects of DCF on VDLCCs and found that these currents were abolished by DCF (298 μM) (**Figure 3B**, lower panel). As indicated in the current–voltage curves (**Figure 3C and D**), DCF significantly decreased the maximal amplitude of VDLCCs. These data strongly suggest that DCF can block the VDLCCs, which interrupts extracellular Ca^2+^ influx and inhibits the rise of [Ca^2+^]_i_, leading to relaxation.

**Figure 4:**
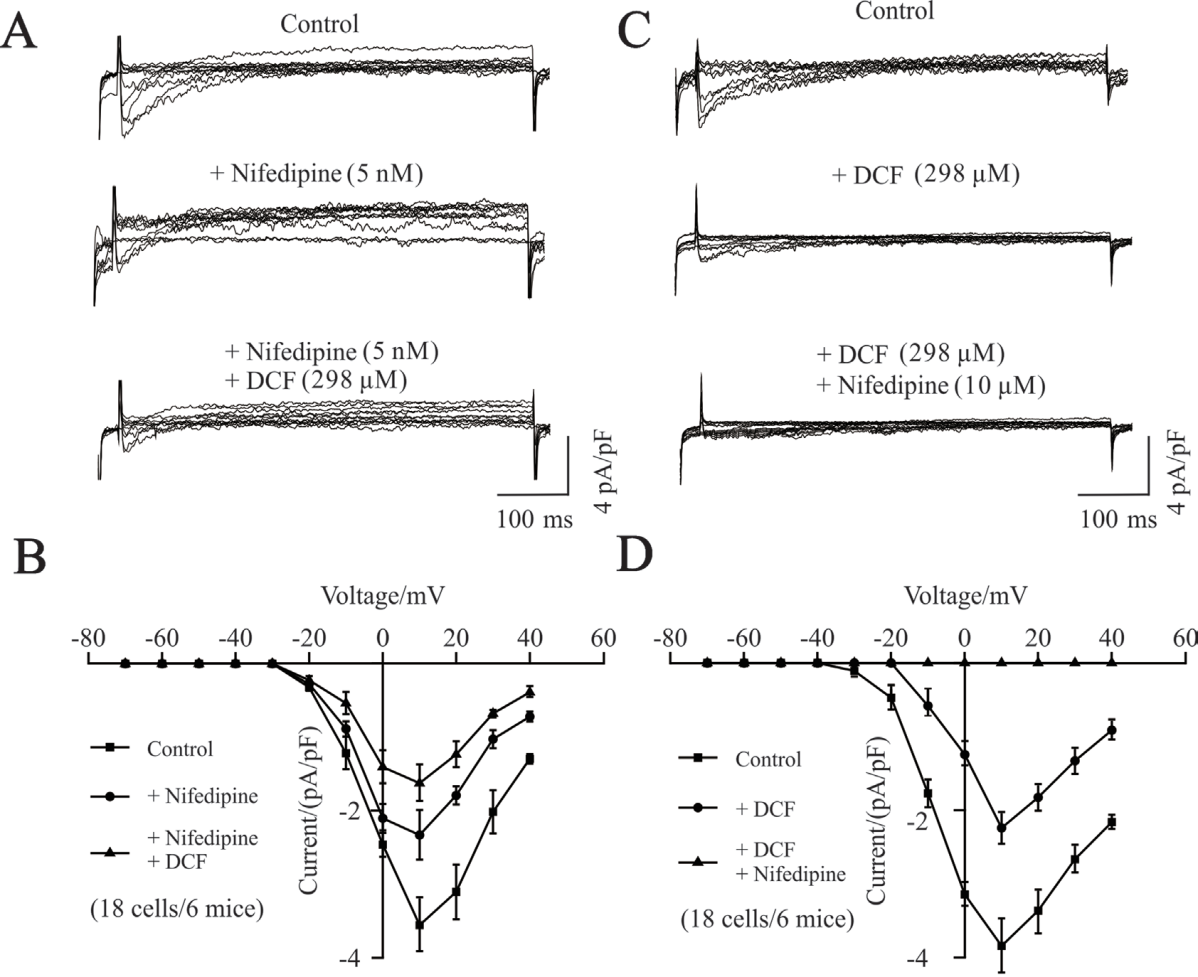
Competitive inhibitory effects of DCF on VDLCCs. **(A)** The patch-clamping technique was used to record the whole-cell VDLCC currents in single ASM cells (upper). Representative VDLCC currents recorded in the presence of a submaximal concentration of nifedipine (5 nM) (middle), while the remaining VDLCC currents were mostly inhibited by 298 μM DCF (lower). **(B)** Statistical analysis of the inhibitory effects of DCF on VDLCC currents based on **(A)** (paired t-test, 18 cells/6 mice). *: *P* < 0.05; **(C)** VDLCC currents were elicited and recorded using the whole-cell patch-clamping technique (upper). Representative VDLCC currents recorded in the presence of a submaximal concentration of DCF (298 μM) (middle), while the remaining VDLCC currents were mostly inhibited by 10 μM DCF (lower). **(D)** Statistical analysis of the inhibitory effects of DCF and nifedipine on VDLCC currents based on **(C)** (paired t-test, 18 cells/6 mice). *: *P* < 0.05. These results suggest that DCF can competitively inhibit VDLCCs.

To further investigate the potential target of DCF, the competitive inhibitory effects of DCF on VDLCC currents were recorded using the whole-cell patch-clamping technique. As indicated in **Figure 4**, the VDLCC currents were elicited and partially inhibited by 5 nM nifedipine. The remaining VDLCC currents were mostly inhibited by 298 μM DCF. Similarly, when the VDLCC currents were partially inhibited by 298 μM DCF, the remaining currents were completely inhibited by 10 μM nifedipine. These data further suggest that DCF can block VDLCCs.

### Diclofenac Sodium Relaxes ACh-Precontracted Tracheal Rings

**Figure 5:**
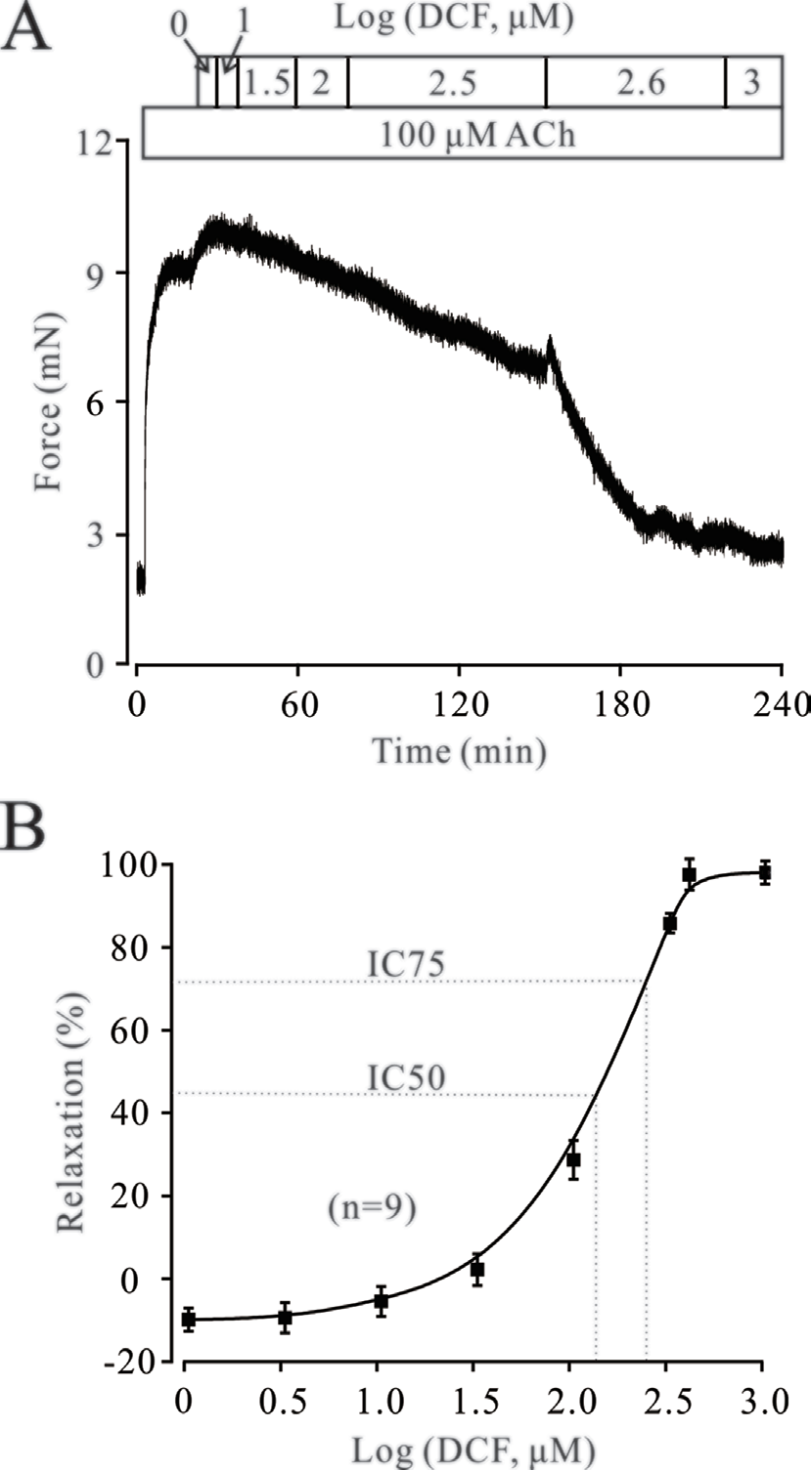
DCF relaxes ACh-precontracted TRs. **(A)** Mouse TRs were precontracted by 100 μM ACh. When the contraction reached steady-state, DCF was cumulatively added into the perfusion bath and induced a relaxation. **(B)** Response-concentration curve for DCF in (A) (n = 9). These data indicate that DCF can relax TRs precontracted by ACh.

The neurotransmitter ACh plays a pivotal role in regulating the contractile state of ASMs. Therefore, we next explored the effects of DCF on ACh-precontracted mouse TRs. As shown in **Figure 5**, 100 μM ACh elicited a large, stable contraction in a mouse TR. This contraction was also inhibited by DCF in a dose-dependent manner (IC75 = 230 μM). These results demonstrate that DCF can relax ACh-precontracted TRs.

### Other than Voltage-Dependent L-Type Ca^2+^ Channels, Diclofenac Sodium Can Still Relax ACh-Precontracted Tracheal Rings

**Figure 6:**
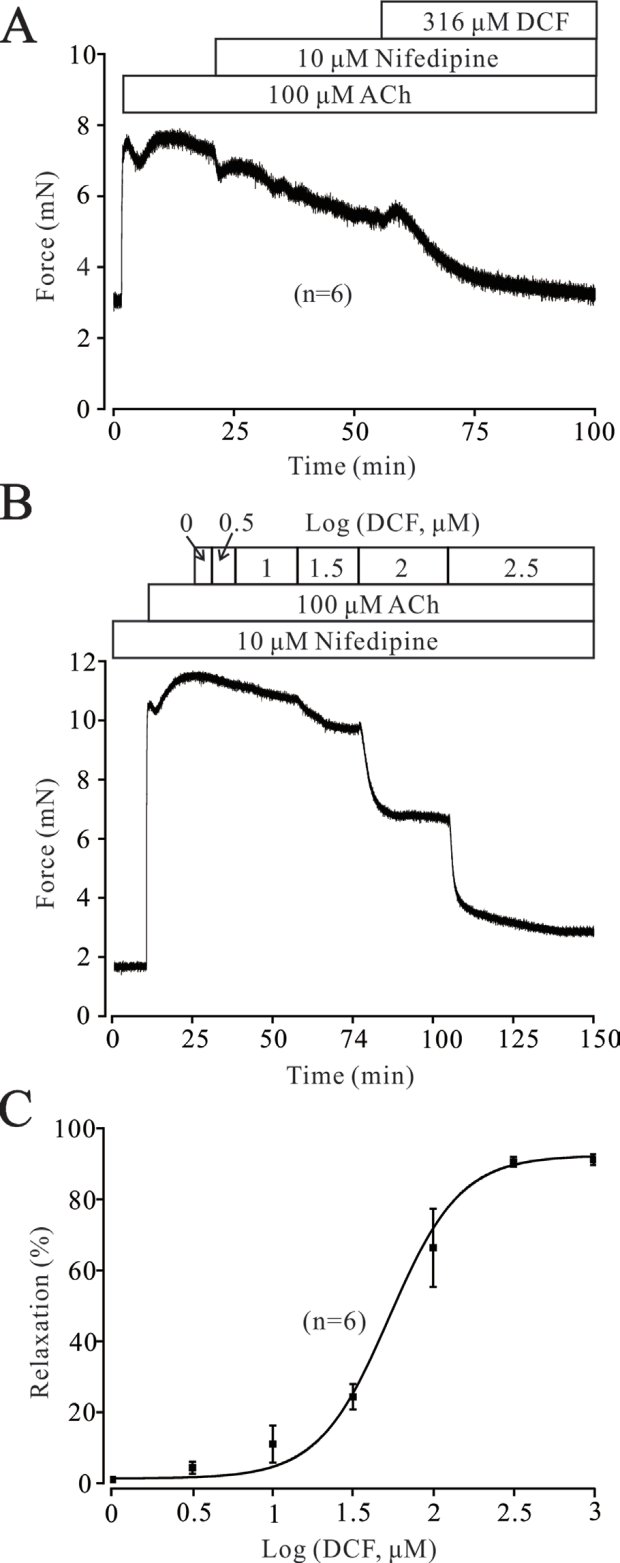
Except for VDLCCs, DCF still relaxes ACh-precontracted TRs. **(A)** ACh-induced contraction was partly inhibited by 10 μM nifedipine. The remaining contraction was mostly inhibited by 316 μM DCF (n = 6). **(B)** With the presence of 10 μM nifedipine, ACh still induced a large stable contraction in mouse TRs. Then, DCF was cumulatively added, which led to a dose-dependent relaxation in the TRs. **(C)** Dose–relaxation curve for DCF in **(B)** (n = 6). These results demonstrate that except for VDLCCs, DCF can relax TRs precontracted by ACh.

Both VDLCCs and NSCCs play important roles in ACh-induced contraction of TRs. As shown in **Figure 6A**, the contraction induced by 100 μM ACh was partly inhibited by 10 μM nifedipine, while the remaining contraction was completely relaxed by 316 μM DCF. Similarly, 100 μM ACh still elicited a large contraction in the TRs preincubated with nifedipine. This contraction was also relaxed by DCF in a dose-dependent manner with the presence of nifedipine (**Figure 6B and C**). These data indicate that DCF can relax TRs in regulating the signaling pathways other than VDLCCs.

### Diclofenac Sodium Inhibits Intracellular Ca^2+^ Release Induced by ACh

**Figure 7:**
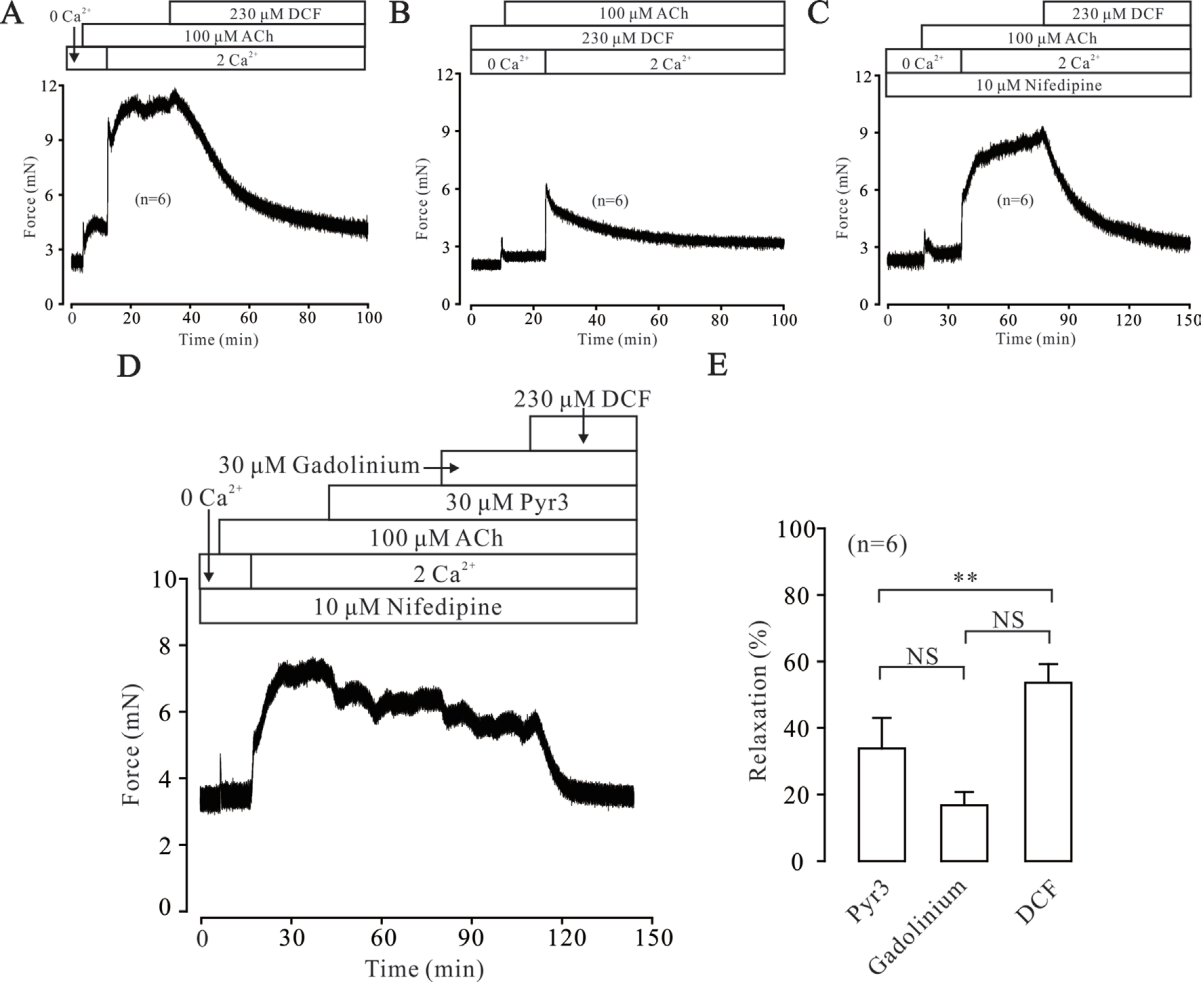
DCF inhibited ACh-induced Ca^2+^ release. **(A)** Under the 0 Ca^2+^ conditions (0 Ca^2+^ + 0.5 mM EGTA), 100 μM induced a small contraction. After the restoration of 2 mM Ca^2+^, a large stable contraction occurred. This contraction was relaxed by 230 μM DCF (n = 6). **(B)** Under 0 Ca^2+^ conditions, 230 μM did not alter the basal tone of mouse TRs. Then, the addition of 100 μM ACh elicited a small contraction. After the restoration of 2 mM Ca^2+^, a larger contraction occurred and was gradually inhibited by DCF (n = 6). **(C)** Under 0 Ca^2+^ conditions, the addition of 10 μM nifedipine did not change the basal tone of mouse TRs. Then, 100 μM ACh was added and induced a very small contraction. After the restoration of 2 mM Ca^2+^, a large stable contraction occurred. Following the addition of 230 μM DCF, the stable contraction was mostly inhibited (n = 6). **(D)** With the presence of 10 μM nifedipine, 100 μM ACh elicited a transient small contraction under 0 Ca^2+^ conditions. Following the restoration of 2 mM Ca^2+^, a large stable contraction occurred, which was partly inhibited by 30 μM Pyr3 and 30 μM gadolinium. The remaining contraction was mostly relaxed by 230 μM DCF (n = 6). **(E)** Summary of relaxant effects of different drugs in **(D)** (n = 6) (paired t-test) **: *P* < 0.01. These data suggest that DCF can inhibit intracellular Ca^2+^ release induced by ACh.

Intracellular Ca^2+^ also plays an important role in the contraction of TRs. Therefore, we explored the changes of [Ca^2+^]_i_ in DCF-treated TRs. As shown in **Figure 7A**, under 0 Ca^2+^ conditions, 100 μM ACh induced a sustained small contraction. After the restoration of 2 mM Ca^2+^, a large contraction occurred and was mostly relaxed by 230 μM DCF. Similarly, under 0 Ca^2+^ conditions, 230 μM DCF did not induce a contraction in a TR, while 100 μM DCF induced a transient small contraction. After the restoration of 2 mM Ca^2+^, a transient large contraction was observed (**Figure 7B**). Meanwhile, with the presence of 10 μM nifedipine, the identical experiment as shown in **Figure 6A** was performed. As indicated in **Figure 7C**, the results were almost the same as that observed in **Figure 6A**, except that the small contraction induced by 100 μM ACh under 0 Ca^2+^ conditions was completely eliminated. Furthermore, the same experiment was performed as in **Figure 6C**; 230 μM DCF still completely relaxed the precontracted TRs, except that the Ca^2+^ sensitization pathways and NSCCs were inhibited by 30 μM Pyr3 and 30 μM gadolinium, respectively (**Figure 7D and E**). These results demonstrate that DCF inhibits intracellular Ca^2+^ release in ASM cells.

### Diclofenac Sodium Inhibits Na^+^/Ca^2+^ Exchange

**Figure 8:**
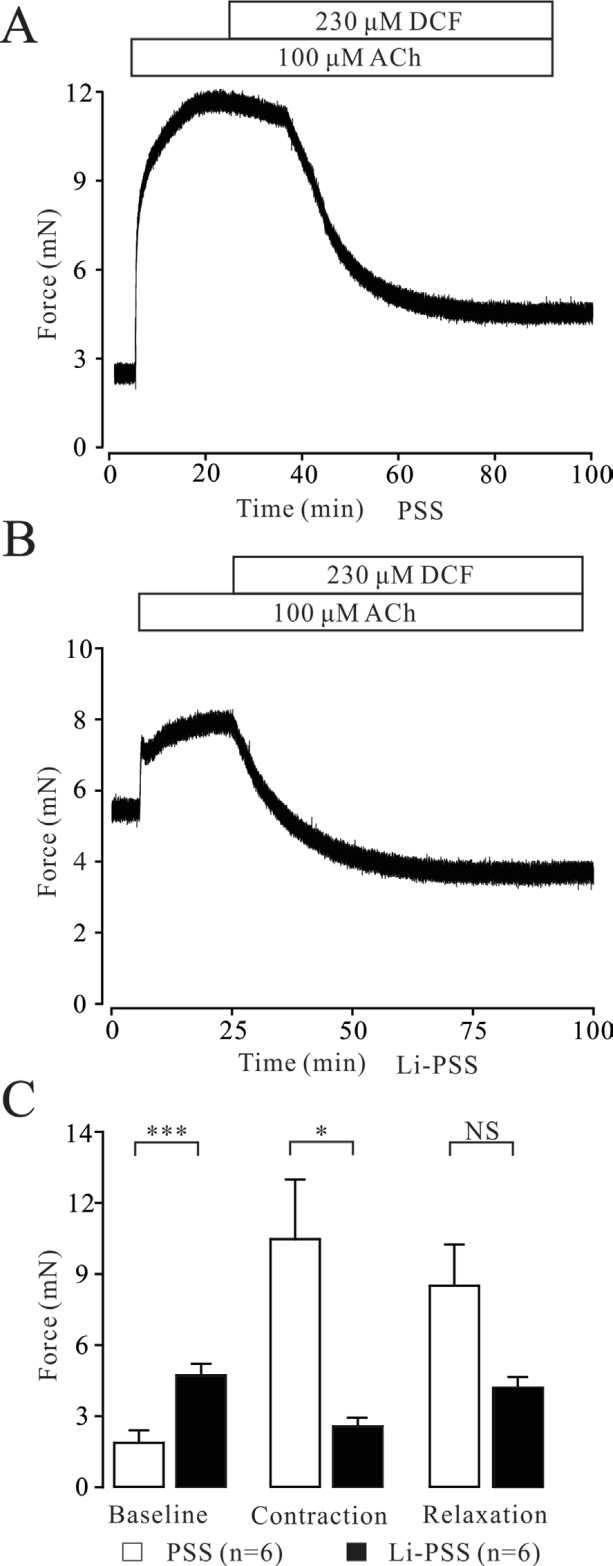
DCF inhibits Na^+^/Ca^2+^ exchange. **(A)** In normal PSS with Na^+^, 100 μM ACh elicited a large contraction in mouse TRs. This contraction was mostly relaxed by 230 μM DCF. **(B)** In Li-PSS (Li^+^ replaced Na^+^) without Na^+^, the basal tone of TRs was significantly increased compared with that of PSS group (*P* < 0.001). The addition of 100 μM ACh elicited a small contraction, which was significantly smaller than that in the PSS group. This small contraction was completely relaxed by 230 μM DCF. **(C)** Effects of Na^+^ on the basal tone, contraction, and relaxation observed in **(A)** and **(B** (n = 6). *: *P* < 0.05; ***: *P* < 0.001. These results indicate that DCF inhibits Na^+^/Ca^2+^ exchange.

Na^+^/Ca^2+^ exchange can affect the intracellular Ca^2+^ concentration, which regulates the contraction of TRs. Therefore, the effects of DCF on Na^+^/Ca^2+^ exchange in TRs were explored. As demonstrated in **Figure 8A**, in normal PSS, 230 μM DCF relaxed the contraction elicited by 100 μM ACh. In Na^+^-free PSS, the basal tone of TRs was significantly higher than that in the PSS group (*P* < 0.001). The addition of 100 μM ACh elicited a small contraction, which was significantly smaller than that in the PSS group (*P* < 0.05) and completely relaxed by 230 μM DCF (**Figures 8B and C**). These data indicate that DCF can relax precontracted TRs via inhibiting Na^+^/Ca^2+^ exchange.

### Diclofenac Sodium Inhibits Nonselective Cation Channel Currents

**Figure 9:**
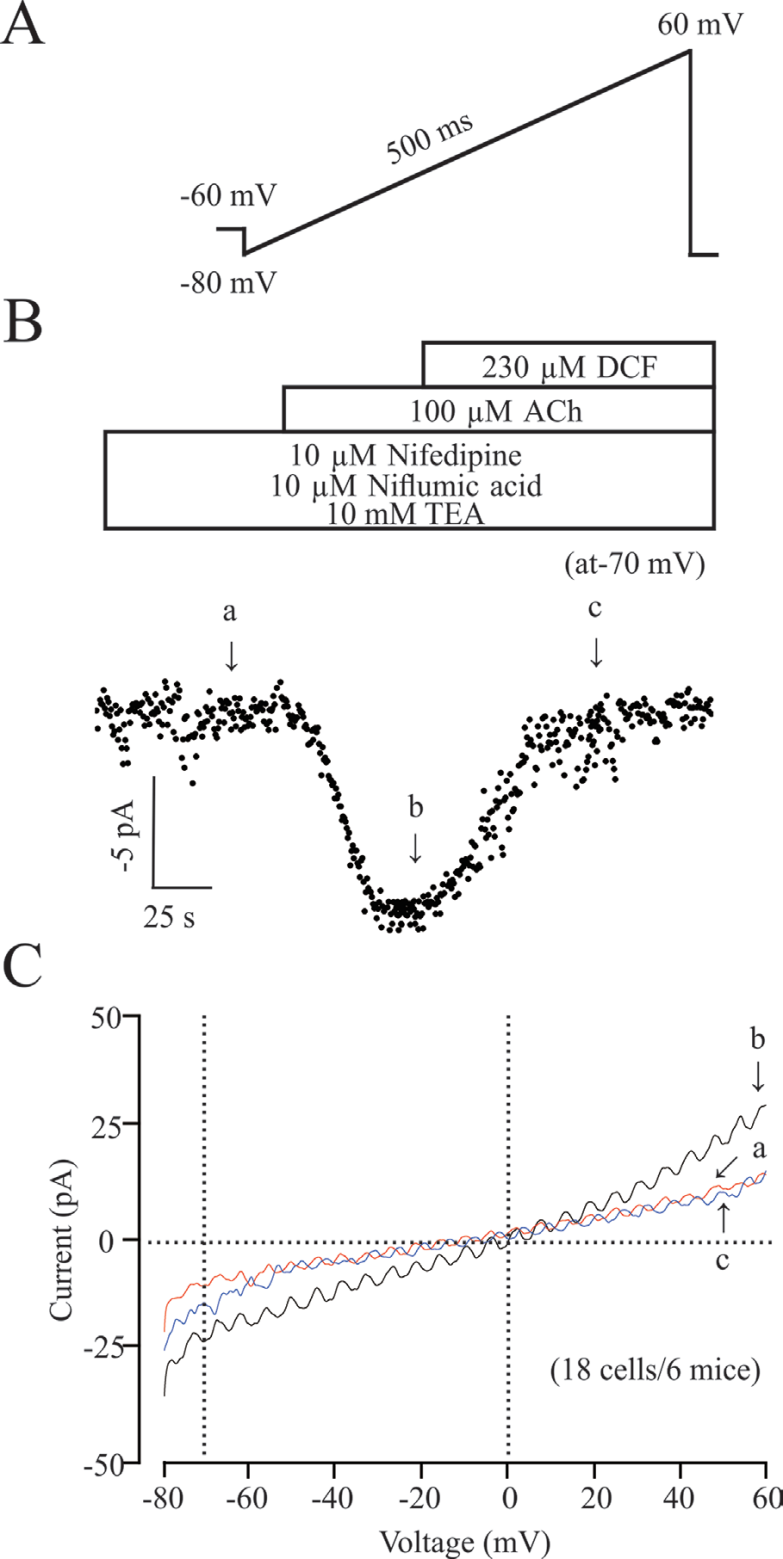
DCF inhibits NSCC currents. **(A)** The ramp performed in recording the NSCC currents. **(B)** With the presence of 10 μM nifedipine, 10 μM niflumic acid, and 10 mM TEA, (inhibitors for VDLCC currents, Cl^−^ currents, and K^+^ currents, respectively), 100 μM ACh induced NSCC currents, which were blocked by 230 μM DCF. **(C)** Representative recordings of net ramp currents at time points b and c (the leakage currents at time point a were subtracted). The average currents at time points b and c at −70 mV (18 cells/6 mice). These results demonstrated that DCF can inhibit NSCC currents.

ACh elicits the contraction of ASM via multiple signaling pathways, such as NSCCs, which regulates the concentration of [Ca^2+^]_i_. Therefore, we studied the effects of DCF on NSCCs using a patching-clamp technique. As shown in **Figure 9**, 230 μM DCF significantly inhibited the NSCCs activated by 100 μM ACh. These results suggest that DCF can inhibit NSCC currents.

### ACh Activated K^+^ Channels and BK Channels

**Figure 10:**
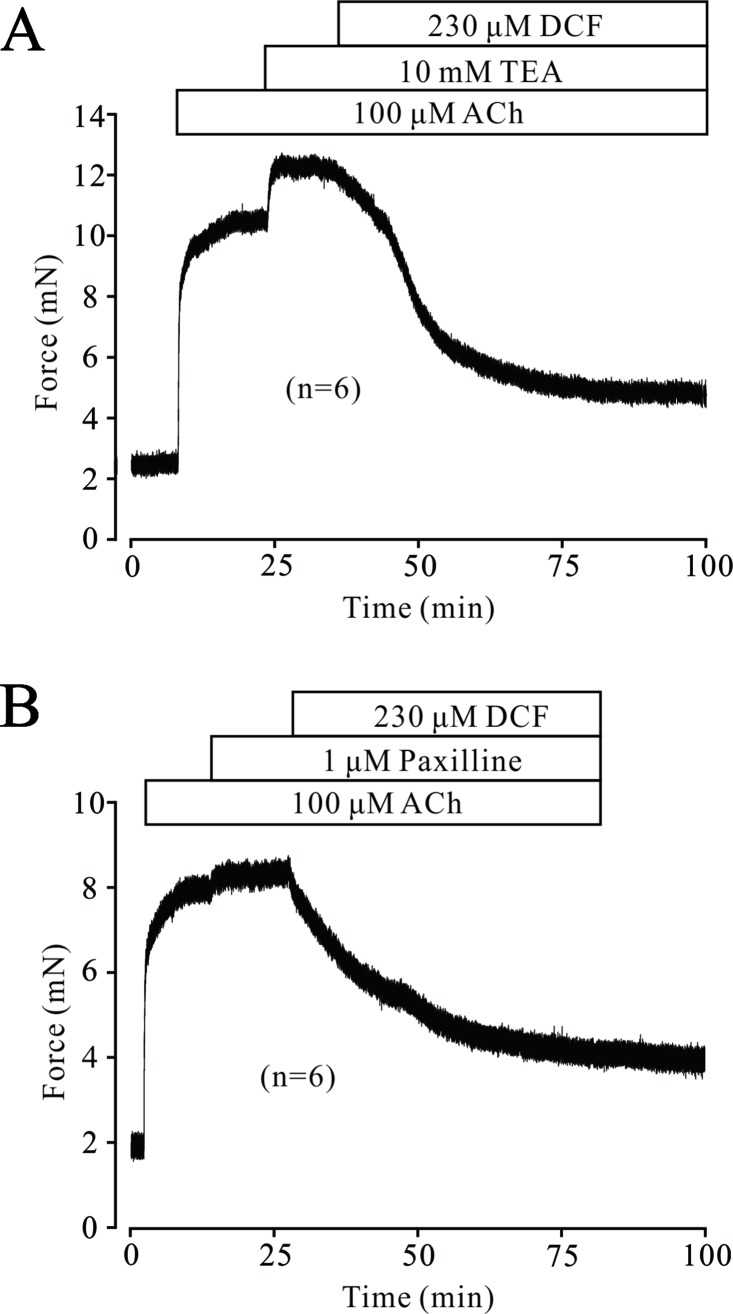
ACh activated K^+^ currents and BK channels. **(A)** A mouse TR was contracted by 100 μM ACh and reached a plateau level. Then, 10 mM TEA was added and enhanced the contraction. However, the large contraction was mostly inhibited by 230 μM DCF (n = 6). **(B)** A large contraction was induced by 100 μM ACh in a mouse TR. This contraction was enhanced by 1 μM paxilline, an inhibitor of BK channels. Similarly, the enhanced contraction was also mostly relaxed by 230 μM DCF (n = 6). These results demonstrate that K^+^ channels, especially BK channels, participate in ACh-induced contraction and may be inhibited by DCF.

In ASM cells, K^+^ channels are ubiquitously distributed and play a pivotal role in regulating the tone of TRs. Next, we tested whether K^+^ channels, especially BK channels, were involved in DCF-induced relaxation. As demonstrated in **Figure 10A**, 100 μM ACh elicited a large stable contraction in a TR, which was enhanced by 10 mM TEA, an inhibitor of K^+^ channels. The enhanced contraction was mostly inhibited by 230 μM DCF. Similarly, a large contraction was also induced by 100 μM ACh and enhanced by 1 μM paxilline, a specific inhibitor of BK channels. The enhanced contraction was still mostly relaxed by DCF (**Figure 10B**). These results demonstrate that K^+^ channels, especially BK channels, are involved in ACh-induced contraction of TRs.

### Diclofenac Sodium Enhanced the K^+^ Currents in Airway Smooth Muscle Cells

**Figure 11:**
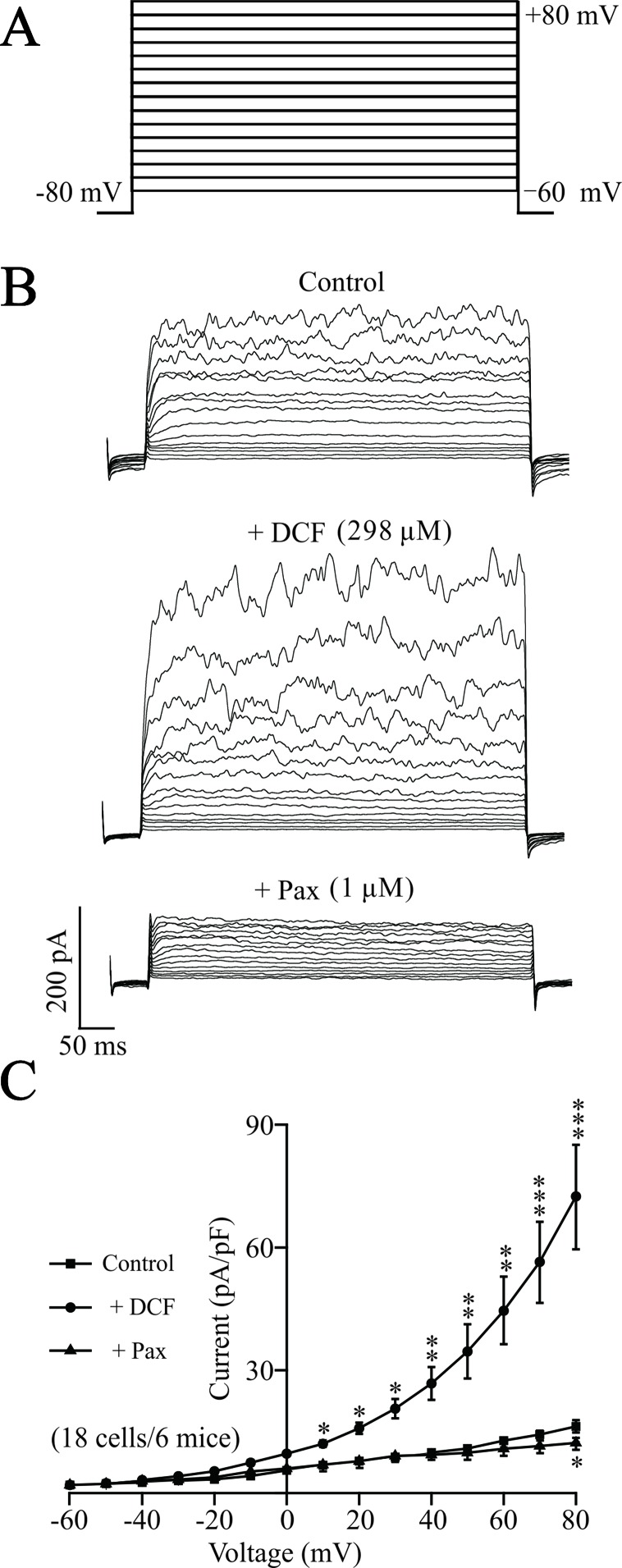
DCF strengthens K^+^ currents in ASM cells. **(A)** The whole-cell recording method was used to record the K^+^ currents. The voltage ranged from −60 to +80 mV at 10-mV increments. **(B)** Representative recording of K^+^ currents under the conditions of control (upper), 298 μM DCF (middle), and 1 μM paxilline (lower) at different voltages. **(C)** Summary of effects of DCF and paxilline on BK currents in **(B)** (18 cells/6 mice) (paired t-test). *: *P* < 0.05; **: *P* < 0.01; ***: *P* < 0.001. These results suggest that DCF can strengthen BK currents.

To elucidate the underlying mechanisms of DCF-induced relaxation, the effects of DCF on K^+^ currents in ASM cells were recorded using the whole-cell patching-clamp technique. As indicated in **Figure 11**, the addition of 298 μM DCF significantly enhanced the amplitudes of K^+^ currents, which was completely inhibited by 1 μM paxilline. The patching-clamp results demonstrate that DCF enhances the amplitude of K^+^ currents in ASM cells.

### Diclofenac Sodium Activates Single BK Channels

**Figure 12:**
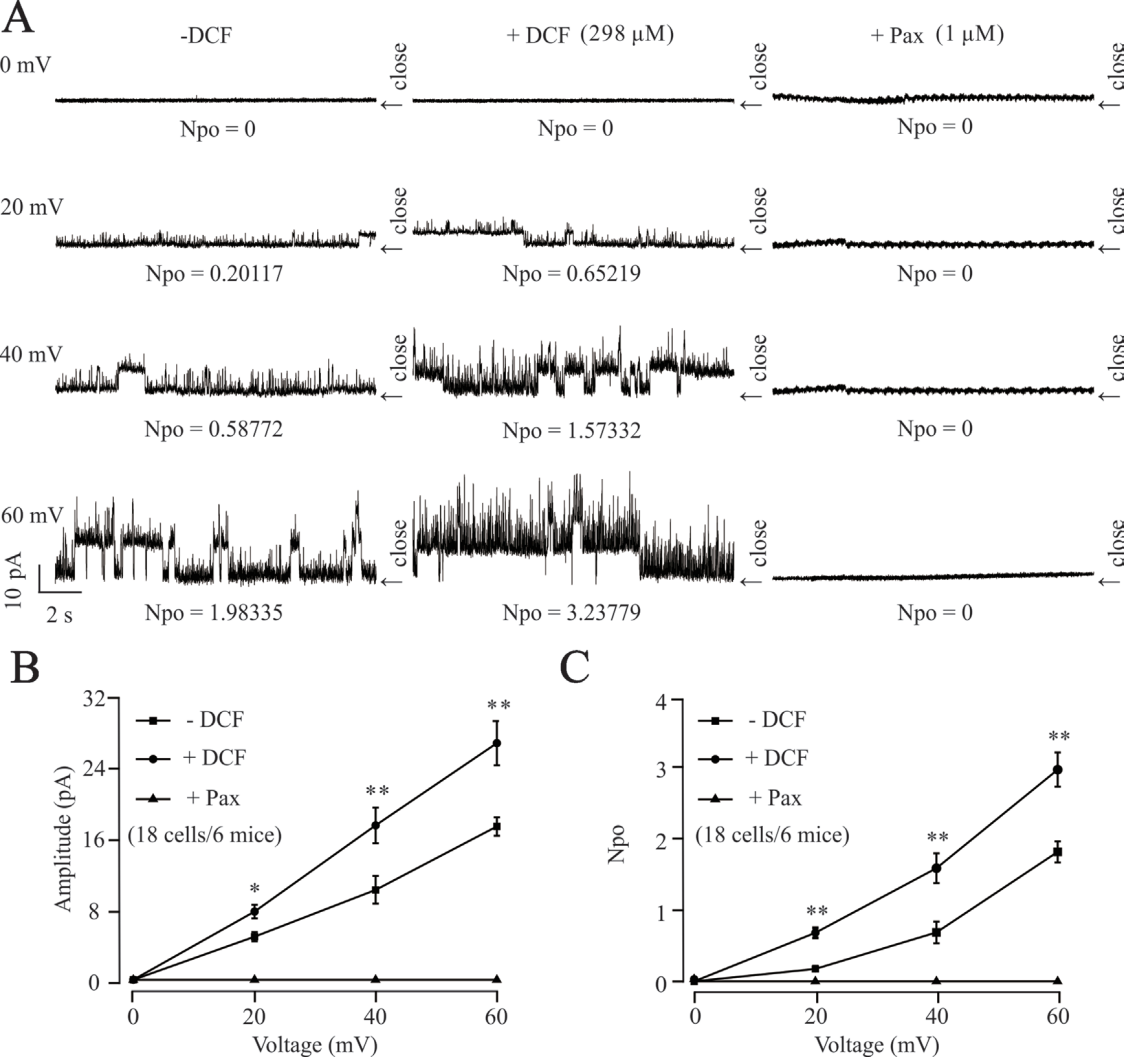
DCF activates single BK channels in mouse ASM cells. **(A)** The outside-out recording method was used to record the single BK channel currents. The amplitudes and opening frequencies of single BK channels were recorded at 0, 20, 40, and 60 mV at control (left), 298 μM DCF (middle), and 1 μM paxilline (right). **(B)** Summary of the amplitudes of single BK channels in **(A)** (18 cells/6 mice) (paired t-test). **(C)** Summary of opening frequencies of single BK channels in **(A)** (paired t-test). *: *P* < 0.05; **: *P* < 0.01; ***: *P* < 0.001. These data indicate that DCF can activate single BK channels.

To further confirm the effects of DCF on K^+^ channels, the changes of single BK channels were recorded using an outside-out patching-clamp technique. As shown in **Figure 12**, the addition of 298 μM DCF significantly increased both the amplitude and the opening frequency of single BK channels. However, the enhanced single BK currents were completely closed by 1 μM paxilline, a known specific inhibitor of BK channels. These data strongly suggest that the single BK channels in ASM cells are enhanced by DCF.

### Diclofenac Sodium Exerts No Harmful Effects on the Activity of Tracheal Rings

**Figure 13:**
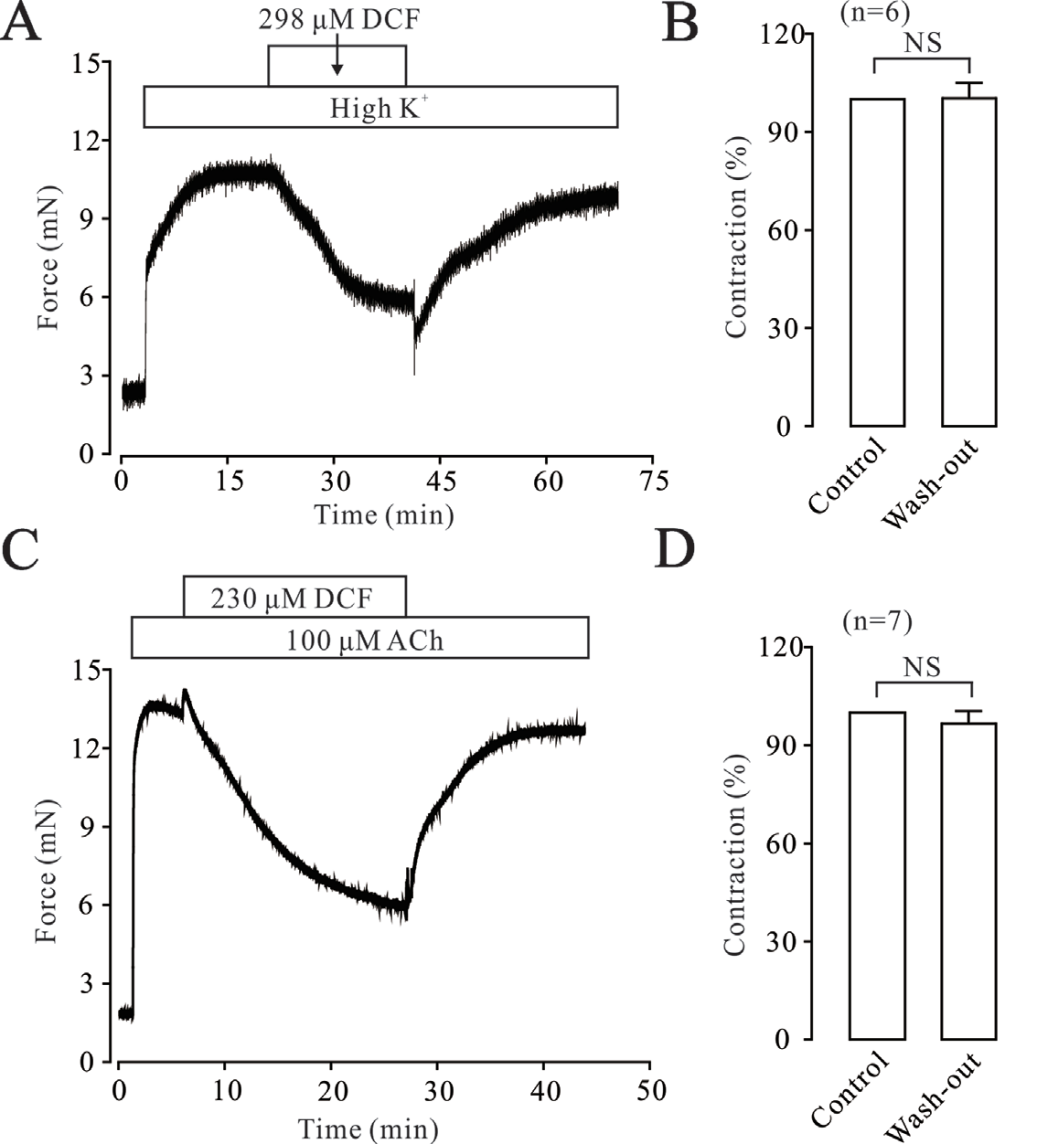
DCF did not alter the activity of mouse TRs. **(A)** High K^+^ induced a large contraction in a mouse TR, which was mostly inhibited by 298 μM DCF. However, washout of DCF led to an obvious increase of contraction in the TR. **(B)** Summary of the effects of DCF and washout on contraction forces in mouse TRs (n = 7) (paired t-test) NS: no significance. **(C)** The contraction induced by 100 μM ACh was mostly inhibited by 230 μM DCF. Similarly, the washout of ACh resulted in an increased contraction force in the TR precontracted by high K^+^. **(D)** Summary of the effects of DCF and washout on contraction force in mouse TRs precontracted by ACh (n = 7) (paired t-test) NS: no significance. These data demonstrate that DCF does not exert harmful effects on the activity of TRs.

To elucidate the possible harmful effects of DCF on ASMs, the effects of DCF on the contraction tone of TRs were studied. As indicated in **Figure 13A and B**, high K^+^ induced a big contraction, which was relaxed by 298 μM DCF. After the washout of DCF, the contraction returned to the control level. Similarly, in **Figure 13C and D**, the same experiments were performed in ACh-precontracted TRs. After the washout of DCF, the contraction was restored to the initial level. These results demonstrate that DCF has no harmful effects on the activity of TRs.

### Diclofenac Sodium Reduced Respiratory System Resistance

**Figure 14:**
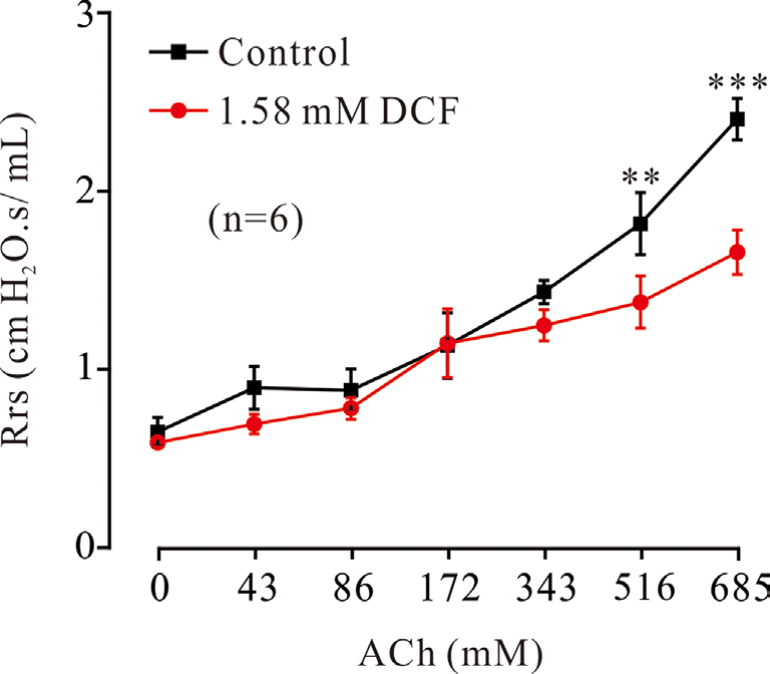
Effects of DCF on Rrs in mice. Mice were anesthetized, and the forced oscillation technique was used to record the Rrs. The parameters for the forced oscillation technique were normalized to baseline at each concentration of aerosolized ACh in control and DCF in experimental group. All values were normalized to individual baseline and expressed as the means ± SEM. **: *P* < 0.01. ***: *P* < 0.001.

To investigate the relaxant effects of DCF on ASMs *in vivo*, the forced oscillation technique was used to record the changes of Rrs. As shown in **Figure 14**, ACh induced increases of Rrs in mice. The increased Rrs were reduced by 1.58 mM DCF. In particular, DCF significantly reduced the increased Rrs induced by ACh at high concentrations.

## Discussion

In this study, we have found that DCF, a common NSAID drug, significantly relaxed high K^+^-/ACh-evoked contractions of mouse TRs in a dose-dependent manner. DCF-induced relaxation is due to the inhibition of VDLCC-mediated Ca^2+^ influx, NSCCs, and Na^+^/Ca^2+^ exchange, resulting in the decrease of [Ca^2+^]_i_ and the relaxation of TRs. Meanwhile, the relaxant effects of DCF in TRs also contributed to the activation of K^+^ channels and BK channels. These results strongly suggest that DCF has the potential ability to relieve bronchospasm.

The contraction of ASMs is regulated by multiple signaling pathways, including intracellular Ca^2+^ release, extracellular Ca^2+^ influx, Ca^2+^ sensitization, Na^+^/Ca^2+^ exchange, and BK channels. Different agonists induce the contraction of ASMs via activating different signaling pathways. For example, high K^+^ mainly depolarizes the cell membrane of ASM cells and leads to the opening of VDLCCs, thereby inducing the contraction of ASMs. ACh exerts its contractile effects via releasing Ca^2+^, activating VDLCCs, and suppressing BK channels. To simulate the multiple pathogenic factors for asthma, two different agonists, high K^+^ and ACh, were used to trigger the contraction of ASMs. The results of muscle force experiments in **Figures 1** and **5** demonstrated that DCF can relax both high K^+^- and ACh-evoked contractions in ASMs.

Nifedipine, as a Ca^2+^ channel blocker, has been shown to block the contraction of ASMs induced by a variety of agonists, including carbachol, prostaglandin F_2α_, histamine, and potassium (Coburn, 1977; Kitamura and Ishihara, 1980). Drazen et al. (1983) reported that nifedipine directly inhibited the constriction of human trachealis muscle. However, in this study, the patch-clamping results in **Figures 3** and **4** suggest that DCF impedes the contraction of ASMs via the competitive inhibition of transmembrane calcium channel VDLCCs, thereby uncoupling the translation of pharmacological/electrical stimulation into mechanical contraction (Coburn, 1977). However, DCF did not completely inhibit the VDLCCs, suggesting that some other signaling pathways are involved in DCF-induced relaxation.

Gadolinium and Pyr3 are inhibitors of NSCCs, which are also involved in the regulation of ASM tones (Halaszovich et al., 2000; Shigeki et al., 2009). The results of muscle force experiments in **Figure 6** suggest that NSCCs participate in ACh-induced contraction in ASMs. The involvement of NSCCs was further confirmed by the patch-clamping recording in **Figure 9**, which was mostly inhibited by 230 μM DCF. These data suggest that DCF can inhibit NSCCs, which in turn results in the decrease of [Ca^2+^]_i_ and leads to relaxation in ASMs.

ACh-evoked contraction in ASMs is generally triggered by increased [Ca^2+^]_i_, which is regulated by both intracellular Ca^2+^ release and extracellular Ca^2+^ influx (Reyes-Garcia et al., 2018). Under the 0 Ca^2+^ conditions, ACh still elicited a small contraction in ASMs with the presence of 230 μM DCF (**Figure 7B** **)**, suggesting that DCF can partly inhibit the release of intracellular Ca^2+^, which leads to the increase in [Ca^2+^]_i_ and relaxation of ASMs.

Na^+^/Ca^2+^ exchange is also involved in the regulation of [Ca^2+^]^­^_i_ in ASM cells (Sathish et al., 2011; Rahman et al., 2012; Sommer et al., 2017). Thus, we further tested the effects of DCF on Na^+^/Ca^2+^ exchange in this process. We found that when the Na^+^ in PSS was replaced by Li^+^, the maximal tone of ACh-induced contraction in mouse TRs significantly decreased, suggesting that Na^+^/Ca^2+^ exchange is involved in ACh-induced contraction. However, DCF can relax ACh-precontracted TRs with or without Na^+^, suggesting Na^+^/Ca^2+^ exchange played a role in DCF-induced relaxation of ASMs. This finding is in line with the previous study reporting that Na^+^/Ca^2+^ exchange was involved in DCF-induced spasmolytic effect in vanadate-induced contraction of rat uterus, where DCF acted via inhibition of calmodulin (Perez Vallina et al., 1995). These results further confirm that DCF can interrupt Na^+^/Ca^2+^ exchange, which inhibits Ca^2+^ influx and decreases the concentration of [Ca^2+^]_i_.

BK channels, large conductance Ca^2+^-activated K^+^ channels, are highly expressed in ASM cells and participate in the regulation of ASM contraction via stabilizing the cell membrane at negative potentials (Zhou et al., 2008). To further explore the mechanisms of DCF-induced relaxation in ASMs, the changes in both K^+^ channels and BK channels were studied. The results of muscle tension measurement experiments in **Figure 10** demonstrated that both K^+^ channels and BK channels contributed the ACh-evoked contraction of mouse ASMs, which was mostly relaxed by DCF. This suggests that DCF may relax ASMs via the regulation of K^+^ and BK channels. This hypothesis was confirmed by the following patch-clamping experiments. The amplitudes of K^+^ currents were significantly enhanced by DCF (**Figure 11**), while both the amplitude and the opening frequencies of single BK channels were markedly strengthened (**Figure 12**). These results demonstrate that DCF can relax ASMs via the activation of BK channels.

Although DCF exhibits excellent relaxant ability in ASMs, as mentioned above, the potential side effects on ASMs remain unclear. Therefore, we investigated the effects of DCF on the activity of ASMs. We found that the maximal contraction forces of ASMs treated by DCF showed no significant differences compared with those of their untreated counterparts (**Figure 13**). These results indicate that DCF does not exert harmful side effects on the activity of ASMs. However, previous studies reported DCF could result in side effects such as renal damage and gastrointestinal damage (Warner et al., 1999; Schmidt et al., 2018). The use of polymer-based nanoparticles such as poly (lactic acid) (PLA) and poly (D, L glycolide), which have high hydrolysis rates, tissue compatibility, and low toxicity, will alleviate the possible side effects of DCF. Meanwhile, in this study, we have mouse TRs with intact epithelium, which plays an important role in regulating ASM tones. The epithelium modulates the contractility of ASMs in diphasic regulatory manners in different ways. For example, it can act as an osmotic sensor through the release of epithelium-derived relaxing factor (Fedan et al., 1999), as a diffusion barrier of ASMs to bronchoactive agents (Gao and Vanhoutte, 1994), or as a regulator of the release of endogenous gamma-amino butyric acid to regulate the ASM tones (Gallos et al., 2013). Further investigations are needed to elucidate the role of the epithelium in DCF-induced relaxation in ASMs.

## Conclusions

The present study demonstrated that DCF has the ability of relaxing precontracted ASMs. This relaxant effect is due to the inhibition of Ca^2+^ release, VDLCC-mediated Ca^2+^ influx, Na^+^/Ca^2+^ exchange, and the enhancement of BK-mediated K^+^ conductance. These findings provide direct evidence that DCF is a new candidate for bronchodilators in curing obstructive respiratory diseases such as asthma and chronic obstructive pulmonary disease.

## Ethics Statement

This study was carried out in accordance with the recommendations of the Institutional Animal Care and Use Committee of the South-Central University for Nationalities guidelines. The protocol was approved by the Animal Ethical Committee of South-Central University for Nationalities (Approval No. 2017-JHS-1).

## Author Contributions

CFC, YY, SS, and SH performed the experiments. MY wrote the manuscript. CLC and QL analyzed the data. JS designed the experiments and revised the manuscript.

## Funding

This work was supported by the National Natural Science Foundation of China (Grant No. 31771274), “the Fundamental Research Funds for the Central Universities,” South-Central University for Nationalities (Grant No. CZY19020), and fund for Key Laboratory Construction of Hubei Province (Grant No. 2018BFC360).

## Conflict of Interest Statement

CL was employed by Wuhan Youzhiyou Biopharmaceutical Co. Ltd. 

The remaining authors declare that the research was conducted in the absence of any commercial or financial relationships that could be construed as a potential conflict of interest.
